# Novel predictors of intravenous immunoglobulin resistance in patients with Kawasaki disease: a retrospective study

**DOI:** 10.3389/fimmu.2024.1399150

**Published:** 2024-07-08

**Authors:** Cong Yi, Yu-Neng Zhou, Jun Guo, Jia Chen, Xiang She

**Affiliations:** Department of Pediatrics, Mianyang Central Hospital, School of Medicine, University of Electronic Science and Technology of China, Mianyang, China

**Keywords:** Kawasaki disease, systemic immune inflammation index, systemic inflammation response index, pan-immune inflammation value, intravenous immunoglobulin resistance

## Abstract

**Objective:**

The aim of this study was to investigate the predictive value of systemic immune inflammation index (SII), systemic inflammatory response index (SIRI), and pan-immune inflammation value (PIV) in predicting intravenous immunoglobulin (IVIG) resistance in children diagnosed with Kawasaki disease (KD).

**Methods:**

The clinical data of pediatric patients diagnosed with Kawasaki disease and admitted to our hospital between January 2006 and December 2022 were retrospectively analyzed.

**Results:**

In total, 771 children diagnosed with KD were included in this study, 86 (11.2%) of whom were diagnosed with IVIG resistance. The correlation between SII, SIRI, PIV and IVIG resistance was evaluated using univariate testing, binary logistic regression analysis, and receiver operating characteristic (ROC) curve analysis. Our study found that the SII, SIRI, and PIV were independent risk factors (p=0.001, p<0.001, and p=0.02, respectively). The area under the ROC curve (AUC) values of the SII, SIRI, and PIV were 0.626 (95% confidence interval (CI): 0.553–0.698, p<0.001), 0.571 (95% CI: 0.500–0.642, p=0.032), and 0.568 (95% CI: 0.495–0.641, p=0.040), respectively, and the cutoff values were 2209.66, 3.77, and 1387.825, respectively.

**Conclusion:**

The SII, SIRI, and PIV have potential value in predicting IVIG resistance in patients with KD.

## 1 Introduction

Kawasaki disease (KD) is an idiopathic vasculitis with non-specific etiology, representing the primary cause of acquired cardiac pathology in pediatric patients. The incidence of KD is predominantly observed in children aged below 5 years, with approximately 25% of untreated cases resulting in the development of coronary artery lesions (1). Currently, it is primarily treated using intravenous immunoglobulin (IVIG) and aspirin (1, 2), the response is excellent; however, 7.5–26.8% of patients do not respond to IVIG, which is defined as IVIG resistance (3–5). Unfortunately, up until now, a definitive cause of IVIG resistance remains elusive. In recent years, the medical community has been dedicated to investigating the risk factors associated with IVIG resistance in KD and resulting in significant advancements. Previous studies have suggested many predictors of IVIG resistance, such as male sex, a C-reactive protein (CRP) level >100 mg/L, a decreased platelet count, and an elevated neutrophil ratio; and a predictive model was developed by these predictors, but it seems to be valid only for East Asian populations (6). Therefore, we hope to find a new indicator that can be widely used in children with KD worldwide.

The quantification of systemic inflammation can be achieved through various biochemical or hematological indicators that are routinely assessed in standard blood tests, or by the ratios derived from these indicators (7). The relationship between neutrophils, lymphocytes, platelets, monocytes, neutrophil-lymphocyte ratio (NLR), and platelet-lymphocyte ratio (PLR) with IVIG resistance in KD has been reported. However, these markers are only implicated in one or two blood routine indicators, whereas KD is a multifaceted systemic inflammatory vasculitis; thus, they fail to comprehensively reflect the inflammatory response associated with KD. In recent years, numerous novel inflammatory markers have been identified in association with inflammatory diseases (8–10). However, there are no studies on the prediction of inflammatory indicators, including the systemic immune inflammation index (SII = platelets × neutrophils/lymphocytes), systemic inflammation response index (SIRI = monocytes × neutrophils/lymphocytes), and pan-immune inflammation value (PIV = monocytes × platelets × neutrophils/lymphocytes), for IVIG non-responsive KD. Thus, the objective of this retrospective study was to explore the potential correlation between these innovative biomarkers and IVIG resistance in children with KD.

## 2 Methods

The Ethics Committee of Mianyang Central Hospital granted approval for this study, which adhered to the principles outlined in the Declaration of Helsinki. Due to its retrospective nature, informed consent requirements were waived (exemption no. S20230322-01). Confidentiality was maintained for all participant data.

### 2.1 Data collection

A total of 917 patients were diagnosed with KD at the Mianyang Central Hospital. We collected detailed data on patients diagnosed with KD, including demographics, clinical indicators, and laboratory parameters. We identified 771 patients with KD and complete data. Diagnoses of KD were made by two independent pediatricians in accordance with the 2017 American Heart Association guidelines (3) and confirmed by a third senior pediatrician if the diagnosis was inconsistent. The definition of complete KD includes a duration of fever for at least 5 days and the presence of at least four out of the following five major clinical features: oral changes, extremity changes, rash, cervical lymphadenopathy, and bilateral bulbar conjunctival injection without exudate. The duration of fever in patients with incomplete KD was ≥5 days, but there were only 2 to 3 main clinical manifestations or even fewer.

All patients were administered intravenous IVIG and oral aspirin. IVIG resistance was defined as recurrent fever with at least one of the main clinical manifestations of KD within 2 weeks (mostly within 2–7 days) after treatment or the body temperature remained higher than 38°C at 36 h after the first dose of IVIG (3). KD shock syndrome (KDSS) was defined as a reduction in systolic blood pressure of ≥20%, or the presence of clinical hypoperfusion (11).

### 2.2 Statistical analysis

We used IBM SPSS Statistics 22.0 for data analysis. The normality assumption was assessed using the Shapiro–Wilk test. The normally distributed continuous variables were presented as mean± standard deviation (mean ± SD), while the non-normally distributed variables were expressed as median (interquartile range, IQR). The t-test or Mann-Whitney U test was employed for intergroup comparisons. Count data were presented as numbers (percentages) and analyzed using the Chi-square test or Fisher’s exact test. Binary logistic regression analysis was employed for conducting multivariate analysis. The receiver operating characteristic (ROC) curve was utilized to assess the statistical significance of laboratory indicators in diagnosing IVIG resistance. The threshold for statistical significance was set at a difference of 0.05.

## 3 Results

In total, 917 patients were diagnosed with KD between January 2006 and December 2022, and there were 551 boys and 366 girls. After excluding patients with incomplete data, 771 (467 boys and 304 girls) were included in this study. Patients were divided into IVIG responders and IVIG non-responders. **Tables 1**, **2** present a summary of the clinical features and laboratory results observed in the patients. In total, 86 (11.15%) patients were IVIG non-responders, including 48 boys and 38 girls. The age distribution and days of IVIG at initiation did not show any statistically significant disparity between the two groups (2.8 ± 2.2 years vs 2.6 ± 2.1 years, p=0.480; and 6.3 ± 2.0 days vs 6.7 ± 1.6 days, p=0.067; respectively). Extremity changes, tachypnea, expectoration, irritability, aseptic meningitis, and KD shock syndrome (KDSS) were significantly more frequent in IVIG non-responders (p=0.018, p<0.001, p=0.044, p<0.001, p<0.001, and p=0.017, respectively). The two groups did not exhibit any significant differences in terms of gender, and the other four diagnostic criteria for complete KD, except extremity changes.

**Table 1:** Clinical Characteristics of Patients with IVIG non-responders and IVIG responders.

Variables	IVIG non-responders	IVIG responders	p value
**Patients, n**	86	685	-
**Age (year), mean ± SD**	2.8 ± 2.2	2.6 ± 2.1	0.480
**gender, male, n (%)**	48 (55.8)	419 (61.2)	0.338
**Days of IVIG at initiation, mean ± SD**	6.3 ± 2.0	6.7 ± 1.6	0.067
**Days of IVIG at initiation<5 days, n (%)**	6 (7.0)	20 (2.9)	0.049*
**Days of IVIG at initiation>10 days, n (%)**	3 (3.5)	26 (3.8)	0.888
**Fever, n (%)**	86 (100)	685 (100)	1.000
**Conjunctival injection, n (%)**	76 (88.3)	613 (89.5)	0.751
**Rash, n (%)**	71 (82.6)	542 (79.1)	0.725
**Oral mucosal changes, n (%)**	74 (86.0)	604 (88.2)	0.568
**Extremity changes, n (%)**	68 (79.1)	455 (66.4)	0.018*
**Cervical lymphadenopathy, n (%)**	58 (67.4)	463 (67.6)	0.978
**Vomiting, n (%)**	18 (20.9)	96 (14.0)	0.222
**Diarrhea, n (%)**	19 (22.1)	169 (24.7)	0.600
**Jaundice, n (%)**	6 (7.0)	24 (3.5)	0.116
**Cough, n (%)**	54 (62.8)	411 (60.0)	0.618
**Expectoration, n (%)**	31 (36.0)	177 (25.8)	0.044*
**Tachypnea, n (%)**	15 (17.4)	11 (1.6)	<0.001*
**Irritability, n (%)**	24 (27.9)	76 (11.1)	<0.001*
**Seizure, n (%)**	1 (1.2)	9 (1.3)	1.000
**Aseptic encephalitis, n (%)**	11 (12.8)	26 (3.8)	<0.001*
**KD shock syndrome, n (%)**	3 (3.5)	3 (0.4)	0.017*
**IKD, n (%)**	14 (16.3)	134 (19.6)	0.466

*p < 0.05.

IKD, incomplete Kawasaki disease; KD, Kawasaki disease; SD, standard deviation.

**Table 2:** Laboratory Findings of Patients with IVIG non-responders and IVIG responders.

Variables	IVIG non-responders	IVIG responders	p value
**WBC (×10 ^9^/L), mean ± SD**	16.45 ± 8.53	16.01 ± 5.83	0.644
**Neutrophils (×10 ^9^/L), mean ± SD**	12.23 ± 7.83	10.98 ± 5.25	0.152
**Lymphocytes (×10 ^9/^L), mean ± SD**	2.87 ± 2.19	3.60 ± 2.00	0.002*
**Monocytes (×10 ^9^/L), mean ± SD**	1.09 ± 0.98	1.09 ± 0.62	0.996
**Platelet (×10 ^9^/L), mean ± SD**	338.7 ± 206.5	341.4 ± 139.5	0.909
**Hemoglobin (g/L), mean ± SD**	102.8 ± 15.6	111.2 ± 12.9	<0.001*
**CRP (mg/L), median (IQR)**	130.0 (74.4-158.4)	86.0 (48.5-140.2)	<0.001*
**ESR (mm/h), median (IQR)**	52.5 (35.0-77.1)	58.0 (41.0-77.0)	0.461
**ALT (IU/L), median (IQR)**	32 (17-66)	27 (15-82)	0.069
**AST (IU/L), median (IQR)**	40 (26-69)	32 (26-48)	0.393
**Serum sodium (mmol/L), mean ± SD**	135.0 ± 3.7	136.7 ± 3.2	<0.001*
**Albumin (g/L), mean ± SD**	32.52 ± 7.35	37.35 ± 5.13	<0.001*
**NLR, median (IQR)**	5.09 (2.51-8.88)	3.11 (1.84-5.42)	<0.001*
**PLR, median (IQR)**	149.17 (84.86-212.83)	101.26 (68.47-145.74)	<0.001*
**SII, median (IQR)**	1539.68 (733.99-2908.71)	977.07 (602.80-1747.49)	<0.001*
**SIRI, median (IQR)**	3.93 (1.78-8.65)	2.87 (1.66-5.45)	0.032*
**PIV, median (IQR)**	1403.32 (540.85-2858.42)	956.29 (489.01-1801.38)	0.04*

*p < 0.05.

ALT, alanine transaminase; AST, aspartate transaminase; CRP, C-reactive proteins; ESR, erythrocyte sediment rate; IQR, interquartile range; NLR, Neutrophil-lymphocyte ratio; PIV, pan-immune inflammation value; PLR, Platelet-lymphocyte ratio; SD, standard deviation; SII, systemic immune inflammation index; SIRI, systemic inflammation response index; WBC, white blood cell count.

In **Table 2**, We also found that lymphocyte, hemoglobin, serum sodium, and albumin levels were significantly lower in IVIG non-responders than in IVIG responders (p=0.002, p<0.001, p<0.001, and p<0.001, respectively). However, CRP, NLR, PLR, SII, SIRI, and PIV were significantly higher in IVIG non-responders than in IVIG responders (p<0.001, p<0.001, p<0.001, p<0.001, p=0.032, and p=0.04, respectively). There were no significant differences in other laboratory parameters between the two groups.

In order to account for all significant variable factors, variables with a significance level of P<0.1 were included in the binary logistic regression analysis, and the corresponding results are presented **Table 3** and in **Supplementary material** (**Supplementary Material Tables 1–5**). The five prediction models were established separately to avoid the collinearity effect among SII, SIRI, PIV, NLR, and PLR. Due to the significant deviation of SII and PIV from other variables, quartile grading was applied to SII and PIV before conducting binary logistic regression analysis. We included the SII in Model 1. Independent risk factors for IVIG resistance include increased SII, tachypnea, and decreased hemoglobin and albumin levels. We included SIRI in Model 2. The independent risk factors for IVIG resistance include increased SIRI levels; tachypnea; and decreased hemoglobin, serum sodium, and albumin levels. We included PIV in Model 3. Independent risk factors for IVIG resistance include increased PIV levels; extremity changes; tachypnea; and decreased days of IVIG at initiation, hemoglobin, serum sodium, and albumin levels. We included NLR in Model 4. Independent risk factors for IVIG resistance include increased NLR levels; and decreased hemoglobin, and albumin levels. We included PLR in Model 5. Independent risk factors for IVIG resistance include increased PLR levels; and decreased days of IVIG at initiation, hemoglobin, and albumin levels. In conclusion, decreased hemoglobin and albumin levels were identified as independent risk factors for IVIG resistance in all five models. In addition, SII, SIRI, PIV, NLR, and PLR were independent risk factors in Model 1, Model 2, Model 3, Model 4 and Model 5, respectively (odds ratio (OR) = 1.51, 95% confidence interval (CI): 1.18–1.92, p=0.001; OR = 1.08, 95% CI: 1.04–1.13, p<0.001; OR = 1.33, 95% CI: 1.06–1.67, p=0.02; OR = 1.09, 95% CI: 1.03–1.015, p=0.002; and OR = 1.004, 95% CI: 1.001–1.006, p=0.002; respectively).

**Table 3:** Binary logistic regression analysis to evaluate risk factors for IVIG resistance in different Models.

Models	Variables	B	S.E.	Waldχ2	OR	95%CI	p value
**Model 1**	Tachypnea	1.22	0.59	4.26	3.38	1.06-10.72	0.04
	Hemoglobin	-0.03	0.01	9.04	0.97	0.95-0.99	0.003
	Albumin	-0.09	0.03	11.36	0.92	0.87-0.96	0.001
	Serum sodium	-0.09	0.04	5.01	0.92	0.85-0.99	0.03
	SII	0.41	0.12	11.01	1.51	1.18-1.92	0.001
**Model 2**	Tachypnea	1.21	0.58	4.30	3.34	1.07-10.46	0.04
	Hemoglobin	-0.04	0.01	11.34	0.97	0.95-0.99	0.001
	Albumin	-0.09	0.03	11.56	0.92	0.87-0.96	0.001
	Serum sodium	-0.09	0.04	5.26	0.92	0.85-0.99	0.02
	SIRI	0.08	0.02	12.96	1.08	1.04-1.13	<0.001
**Model 3**	Days of IVIG at initiation	-0.17	0.08	4.30	0.84	0.71-0.99	0.04
	Tachypnea	1.23	0.58	4.54	3.42	1.10-10.61	0.03
	Hemoglobin	-0.03	0.01	9.03	0.97	0.95-0.99	0.003
	Albumin	-0.09	0.03	13.80	0.91	0.87-0.96	<0.001
	Serum sodium	-0.09	0.04	5.21	0.92	0.85-0.99	0.02
	PIV	0.29	0.12	5.93	1.33	1.06-1.67	0.02
**Model 4**	Tachypnea	1.18	0.59	4.03	3.26	1.03-10.34	0.045
	Hemoglobin	-0.04	0.01	10.78	0.97	0.95-0.99	0.001
	Albumin	-0.08	0.03	9.60	0.92	0.88-0.97	0.002
	NLR	0.09	0.03	9.91	1.09	1.03-1.15	0.002
**Model 5**	Days of IVIG at initiation	-0.17	0.08	4.09	0.85	0.718-0.995	0.04
	Hemoglobin	-0.03	0.01	7.02	0.97	0.95-0.99	0.008
	Albumin	-0.09	0.03	12.28	0.91	0.87-0.96	<0.001
	PLR	0.004	0.001	9.25	1.004	1.001-1.006	0.002

CI, confidence interval; NLR, Neutrophil-lymphocyte ratio; OR, odds ratio; PIV, pan-immune inflammation value; PLR, Platelet-lymphocyte ratio; SII, systemic immune inflammation index; SIRI, systemic inflammation response index.

**Figure 1:**
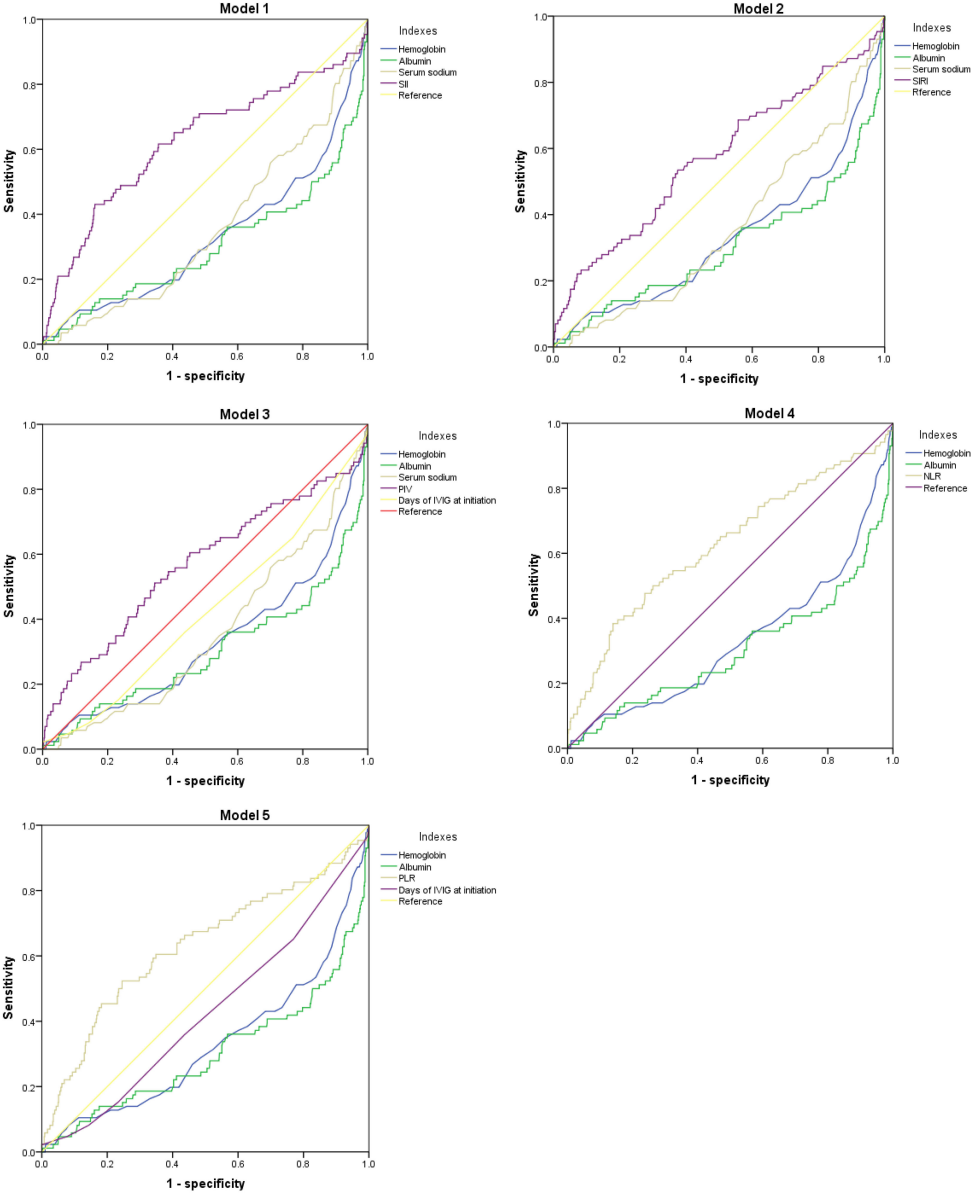
The ROC curve for Models in predicting IVIG resistance KD.

In **Table 4**; **Figure 1**, we revealed that the predictive power of SII, SIRI, PIV, NLR, PLR, hemoglobin, and albumin for IVIG resistant KD achieved an area under the ROC curve (AUC) value of 0.626 (95% CI: 0.553–0.698, p<0.001), 0.571 (95% CI: 0.500–0.642, p=0.032), 0.568 (95% CI: 0.495–0.641, p=0.040), 0.626 (95% CI: 0.557–0.696, p<0.001), 0.629 (95% CI: 0.558–0.700, p<0.001), 0.669 (95% CI: 0.603–0.736, p<0.001), and 0.698 (95% CI: 0.629–0.767, p<0.001), respectively. And the comprehensive table presents the AUCs for all variables included in **Supplementary material** (**Supplementary Material Tables 6–10**). In **Table 5**, we obtained the cutoff values of the SII, SIRI, PIV, NLR, and PLR as 2209.66, 3.77, 1387.825, 7.655, and 147.55 respectively, by calculating the Youden index. Compared with SII, SIRI, PIV, NLR and PLR; hemoglobin and albumin had lower specificity.

**Table 4:** Area under the curve of SII, SIRI, PIV, NLR, and PLR in different Models.

Models	Variables	AUC	95%CI	p value
**Model 1**	SII	0.626	0.553-0.698	<0.001
**Model 2**	SIRI	0.571	0.500-0.642	0.032
**Model 3**	PIV	0.568	0.495-0.641	0.040
**Model 4**	NLR	0.626	0.557-0.696	<0.001
**Model 5**	PLR	0.629	0.558-0.700	<0.001

AUC, area under curve; CI, confidence interval; NLR, Neutrophil-lymphocyte ratio; PLR, Platelet-lymphocyte ratio; PIV, pan-immune inflammation value; SII, systemic immune inflammation index, SIRI, systemic inflammation response index.

**Table 5:** ROC curve for variables’ levels for distinguishing between the IVIG responders and the non-responders.

Models	Variables	Cutoff	Sensitivity (%)	Specificity (%)	PPV (%)	NPV (%)
**Model 1**	SII	2209.66	43.0	62.5	53.4	52.3
**Model 2**	SIRI	3.77	53.5	83.9	76.9	64.3
**Model 3**	PIV	1387.825	51.2	65.5	59.7	57.3
**Model 4**	NLR	7.655	38.4	86.0	73.3	58.3
**Model 5**	PLR	147.55	52.3	75.5	68.1	61.3
**All models**	Hemoglobin	98.5	53.5	16.2	39.0	25.8
**All models**	Albumin	32.805	44.2	18.0	35.0	24.4

NLR, Neutrophil-lymphocyte ratio; PLR, Platelet-lymphocyte ratio; PIV, pan-immune inflammation value; PPV, positive predictive value; NPV, negative predictive value; SII, systemic immune inflammation index, SIRI, systemic inflammation response index.

## 4 Discussion

The issue of IVIG resistance in patients with KD is a significant concern within the medical community. Previous reports indicate that patients who exhibit resistance to IVIG treatment are at an elevated risk for developing coronary artery lesions (3). Pediatricians worldwide have completed a lot of studies to search for risk factors for IVIG resistance in KD. In our study, tachypnea and low hemoglobin and albumin levels were found to be independent risk factors. Recently, Wang et al. (12) developed a new predictive model, in which hemoglobin reduction was an independent risk factor. Previous studies have suggested that low albumin levels is a risk factor for IVIG resistance (6, 13), which is consistent with the results of our study. In our previous study (2), we found that children with KD who had tachypnea had a more severe inflammatory response and were more likely to have damage to other organs, such as the acute abdomen. It has been reported that patients with KD-associated pulmonary involvement (KD-PI) have a higher incidence of IVIG resistance than the KD controls. Meanwhile, compared with KD controls, the patients with KD-PI have significantly high levels of CRP and Procalcitonin (PCT) (14). These suggest that atypical clinical symptoms, such as tachypnea, may be accompanied by a more severe inflammatory response, which often leads to IVIG resistance. The results from Model 3 and Model 5 indicate that early use of IVIG was a significant risk factor for developing resistance to IVIG treatment. It is worth noting that our analysis in **Table 1** revealed a statistically significant difference between the two groups of children who received IVIG within 5 days of disease onset, which aligns with the findings reported by Kobayashi et al. (6).

The SII, SIRI, and PIV were first proposed in 2014, 2016, and 2020, respectively (8–10), and were initially utilized in the domain of cancer-associated inflammation for prognostic prediction. In recent years, they have gradually been applied to other diseases (15–17). So far, only a few studies have described the relationship between SII and KD. The study conducted by Huang et al. (18) revealed that SII serves as a significant risk factor for the development of coronary artery lesions in patients with KD. However, to date, few studies have reported an association between the SII and IVIG resistance in patients with KD. Furthermore, no studies have utilized SIRI and PIV to patients with KD. Previous studies have reported that NLR and PLR are risk factors for IVIG resistance in KD (19–21). In our study, we revealed, for the first time, that the SII, SIRI, and PIV are independent risk factors for IVIG resistance. This may help clinical pediatricians to better predict IVIG-resistant KD patients.

Studies have shown that KD is characterized by acute, non-specific inflammation of the small- and medium-sized arteries; however, its etiology remains unknown (1). The acute phase of KD is characterized by dynamic changes in various inflammatory cells and factors. These inflammatory cells and factors act together to induce inflammation in the systemic arteries, particularly the coronary arteries, ultimately leading to the development of KD (3, 22).

The neutrophils, as the predominant leukocytes in human circulation, play a pivotal role in the innate immune response (23). Elevated neutrophil counts are frequently observed during the acute phase of KD. Reactive oxygen species, neutrophil elastase, and myeloperoxidase released during the inflammatory response will activate neutrophils. Recent studies show that endothelial injury caused by activated neutrophils is involved in the development of KD vasculitis (24, 25). The increased proportion of neutrophils is considered a significant risk factor for IVIG resistance in KD, and it has been widely utilized as a predictive marker for IVIG resistance in this condition (6).

Lymphocytes and monocytes are important subpopulations of white blood cells. Stock et al. (26) reported that intimal macrophages develop from circulating monocytes, and that macrophages infiltrate the inflamed coronary intima through transluminal migration. This may be the mechanism of arteritis in KD. Lymphocytes are essential for maintaining vascular endothelial health (18). Moreover, low lymphocyte is a poor prognostic factor for acute coronary syndromes and heart failure (27, 28). Platelets are formed by the fragmentation of megakaryocytes and play crucial roles in hemostasis, coagulation, and inflammatory responses (29). Platelet counts are usually elevated at 2-3 weeks in KD patients, and this is considered to be benign and reactive response (3). However, later studies suggest that low lymphocyte and platelet counts are risk factors of IVIG resistance in patients with KD (30, 31). The present study conclusively demonstrates the significant involvement of neutrophils, lymphocytes, monocytes, and platelets in the inflammatory process of KD.

The composite inflammatory markers of SII, SIRI, and PIV offer distinct advantages over individual neutrophils, lymphocytes, monocytes, or platelets in providing a comprehensive assessment of inflammation. And compared with other predictors, SII, SIRI, and PIV are simple and easy to obtain by blood routine. Although our study was a single-center retrospective study, these three markers demonstrated significant potential as readily available inflammatory indicators for predicting IVIG resistance in KD patients.

Our study has certain limitations. First, this was a retrospective study conducted at a single center and thus was subject to inherent selection bias. Second, the sample size of this study was small. Third, data were missing for some earlier cases. Therefore, we need a large sample, multi-center prospective study to further confirm the view of this study.

## Data availability statement

The raw data supporting the conclusions of this article will be made available by the authors, without undue reservation.

## Ethics statement

The studies involving humans were approved by The Ethics Committee of Mianyang Central Hospital. The studies were conducted in accordance with the local legislation and institutional requirements. The ethics committee/institutional review board waived the requirement of written informed consent for participation from the participants or the participants’ legal guardians/next of kin because This was a retrospective study.

## Author contributions

CY: Data curation, Formal analysis, Investigation, Project administration, Writing – original draft. YZ: Data curation, Formal analysis, Investigation, Supervision, Writing – original draft. JG: Data curation, Methodology, Supervision, Writing – original draft. JC: Data curation, Software, Supervision, Writing – original draft. XS: Funding acquisition, Supervision, Writing – original draft, Writing – review & editing.
