# Supplementary material for: Novel predictors of intravenous immunoglobulin resistance in patients with Kawasaki disease: a retrospective study
Source: Front Immunol. 2024 Jul 8;15:1399150. doi: 10.3389/fimmu.2024.1399150 (PMC11260624; doi:10.3389/fimmu.2024.1399150)
Supplement: Supplementary file 1 [file DataSheet_1.docx]

**Table 1** Binary logistic regression analysis to evaluate risk factors for IVIG resistance in Model 1.

| Variables | B | S.E. | Waldχ2 | OR | 95%CI | p value |
| --- | --- | --- | --- | --- | --- | --- |
| **Days of IVIG at initiation** | -0.15 | 0.08 | 3.19 | 0.86 | 0.73-1.02 | 0.07 |
| **Extremity changes** | 0.48 | 0.31 | 2.46 | 1.61 | 0.89-2.94 | 0.12 |
| **Tachypnea** | 1.22 | 0.59 | 4.26 | 3.38 | 1.06-10.72 | 0.04* |
| **Expectoration** | 0.24 | 0.29 | 0.71 | 1.27 | 0.73-2.23 | 0.40 |
| **Irritability** | 0.17 | 0.40 | 0.19 | 1.19 | 0.55-2.57 | 0.67 |
| **Aseptic encephalitis** | 0.36 | 0.57 | 0.39 | 1.43 | 0.47-4.34 | 0.53 |
| **KD shock syndrome** | -0.80 | 1.02 | 0.61 | 0.45 | 0.06-3.33 | 0.43 |
| **Hemoglobin** | -0.03 | 0.01 | 9.04 | 0.97 | 0.95-0.99 | 0.003* |
| **CRP** | <0.001 | 0.002 | 0.02 | 1.000 | 0.995-1.004 | 0.88 |
| **ALT** | -0.001 | 0.001 | 0.69 | 0.999 | 0.996-1.002 | 0.41 |
| **Albumin** | -0.09 | 0.03 | 11.36 | 0.92 | 0.87-0.96 | 0.001* |
| **Serum sodium** | -0.09 | 0.04 | 5.01 | 0.92 | 0.85-0.99 | 0.03* |
| **SII** | 0.41 | 0.12 | 11.01 | 1.51 | 1.18-1.92 | 0.001* |

**Note:** *p < 0.05.

**Abbreviations:** ALT, alanine transaminase; CRP, C-reactive proteins; KD, Kawasaki disease; SII, systemic immune inflammation index.

**Table 2** Binary logistic regression analysis to evaluate risk factors for IVIG resistance in Model 2.

| Variables | B | S.E. | Waldχ2 | OR | 95%CI | p value |
| --- | --- | --- | --- | --- | --- | --- |
| **Days of IVIG at initiation** | -0.15 | 0.08 | 3.32 | 0.86 | 0.73-1.01 | 0.07 |
| **Extremity changes** | 0.54 | 0.31 | 3.06 | 1.72 | 0.94-3.16 | 0.08 |
| **Tachypnea** | 1.21 | 0.58 | 4.30 | 3.34 | 1.07-10.46 | 0.04* |
| **Expectoration** | 0.34 | 0.29 | 1.38 | 1.41 | 0.80-2.49 | 0.24 |
| **Irritability** | 0.02 | 0.40 | 0.002 | 1.02 | 0.46-2.24 | 0.97 |
| **Aseptic encephalitis** | 0.34 | 0.57 | 0.36 | 1.41 | 0.46-4.32 | 0.55 |
| **KD shock syndrome** | -0.69 | 1.06 | 0.43 | 0.50 | 0.06-3.99 | 0.51 |
| **Hemoglobin** | -0.04 | 0.01 | 11.34 | 0.97 | 0.95-0.99 | 0.001* |
| **CRP** | 0.000 | 0.002 | 0.01 | 1.00 | 0.996-1.004 | 0.91 |
| **ALT** | -0.001 | 0.001 | 0.62 | 0.999 | 0.996-1.002 | 0.43 |
| **Albumin** | -0.09 | 0.03 | 11.56 | 0.92 | 0.87-0.96 | 0.001* |
| **Serum sodium** | -0.09 | 0.04 | 5.26 | 0.92 | 0.85-0.99 | 0.02* |
| **SIRI** | 0.08 | 0.02 | 12.96 | 1.08 | 1.04-1.13 | <0.001* |

**Note:** *p < 0.05.

**Abbreviations:** ALT, alanine transaminase; CRP, C-reactive proteins; KD, Kawasaki disease; SIRI, systemic inflammation response index.

**Table 3** Binary logistic regression analysis to evaluate risk factors for IVIG resistance in Model 3.

| Variables | B | S.E. | Waldχ2 | OR | 95%CI | p value |
| --- | --- | --- | --- | --- | --- | --- |
| **Days of IVIG at initiation** | -0.17 | 0.08 | 4.30 | 0.84 | 0.71-0.99 | 0.04* |
| **Extremity changes** | 0.46 | 0.31 | 2.28 | 1.58 | 0.87-2.88 | 0.13 |
| **Tachypnea** | 1.23 | 0.58 | 4.54 | 3.42 | 1.10-10.61 | 0.03* |
| **Expectoration** | 0.24 | 0.29 | 0.71 | 1.27 | 0.73-2.23 | 0.40 |
| **Irritability** | 0.11 | 0.39 | 0.08 | 1.11 | 0.52-2.41 | 0.79 |
| **Aseptic encephalitis** | 0.37 | 0.57 | 0.43 | 1.45 | 0.48-4.41 | 0.51 |
| **KD shock syndrome** | -0.67 | 1.01 | 0.44 | 0.51 | 0.07-3.73 | 0.51 |
| **Hemoglobin** | -0.03 | 0.01 | 9.03 | 0.97 | 0.95-0.99 | 0.003* |
| **CRP** | <0.001 | .002 | 0.003 | 1.000 | 0.996-1.004 | 0.96 |
| **ALT** | -0.001 | 0.001 | 0.32 | 0.999 | 0.997-1.002 | 0.57 |
| **Albumin** | -0.09 | 0.03 | 13.80 | 0.91 | 0.87-0.96 | <0.001* |
| **Serum sodium** | -0.09 | 0.04 | 5.21 | 0.92 | 0.85-0.99 | 0.02* |
| **PIV** | 0.29 | 0.12 | 5.93 | 1.33 | 1.06-1.67 | 0.02* |

**Note:** *p < 0.05.

**Abbreviations:** ALT, alanine transaminase; CRP, C-reactive proteins; KD, Kawasaki disease; PIV, pan-immune inflammation value.

**Table 4** Binary logistic regression analysis to evaluate risk factors for IVIG resistance in Model 4.

| Variables | B | S.E. | Waldχ2 | OR | 95%CI | p value |
| --- | --- | --- | --- | --- | --- | --- |
| **Days of IVIG at initiation** | -0.14 | 0.08 | 2.83 | 0.87 | 0.74-1.02 | 0.09 |
| **Extremity changes** | 0.48 | 0.30 | 2.45 | 1.61 | 0.89-2.92 | 0.12 |
| **Tachypnea** | 1.18 | 0.59 | 4.03 | 3.26 | 1.03-10.34 | 0.045 |
| **Expectoration** | 0.24 | 0.29 | 0.70 | 1.27 | 0.72-2.24 | 0.40 |
| **Irritability** | 0.12 | 0.39 | 0.10 | 1.13 | 0.52-2.45 | 0.76 |
| **Aseptic encephalitis** | 0.27 | 0.58 | 0.23 | 1.32 | 0.42-4.07 | 0.64 |
| **KD shock syndrome** | -1.13 | 1.06 | 1.14 | 0.32 | 0.04-2.58 | 0.29 |
| **Hemoglobin** | -0.04 | 0.01 | 10.78 | 0.97 | 0.95-0.99 | 0.001* |
| **CRP** | <0.001 | 0.002 | 0.02 | 1.000 | 0.996-1.004 | 0.90 |
| **ALT** | -0.001 | 0.001 | 1.01 | 0.999 | 0.996-1.001 | 0.32 |
| **Albumin** | -0.08 | 0.03 | 9.60 | 0.92 | 0.88-0.97 | 0.002* |
| **Serum sodium** | -0.07 | 0.04 | 2.97 | 0.94 | 0.87-1.01 | 0.09 |
| **NLR** | 0.09 | 0.03 | 9.91 | 1.09 | 1.03-1.15 | 0.002* |

**Note:** *p < 0.05.

**Abbreviations:** ALT, alanine transaminase; CRP, C-reactive proteins; KD, Kawasaki disease; NLR, Neutrophil-lymphocyte ratio.

**Table 5** Binary logistic regression analysis to evaluate risk factors for IVIG resistance in Model 5.

| Variables | B | S.E. | Waldχ2 | OR | 95%CI | p value |
| --- | --- | --- | --- | --- | --- | --- |
| **Days of IVIG at initiation** | -0.17 | 0.08 | 4.09 | 0.85 | 0.718-0.995 | 0.04* |
| **Extremity changes** | 0.52 | 0.31 | 2.91 | 1.69 | 0.93-3.07 | 0.09 |
| **Tachypnea** | 1.16 | 0.59 | 3.83 | 3.18 | 0.99-10.15 | 0.05 |
| **Expectoration** | 0.16 | 0.29 | 0.30 | 1.17 | 0.67-2.06 | 0.58 |
| **Irritability** | 0.12 | 0.40 | 0.09 | 1.13 | 0.52-2.45 | 0.77 |
| **Aseptic encephalitis** | 0.30 | 0.57 | 0.27 | 1.34 | 0.44-4.08 | 0.60 |
| **KD shock syndrome** | -0.71 | 1.06 | 0.45 | 0.49 | 0.06-3.89 | 0.50 |
| **Hemoglobin** | -0.03 | 0.01 | 7.02 | 0.97 | 0.95-0.99 | 0.008* |
| **CRP** | 0.001 | 0.002 | 0.30 | 1.001 | 0.997-1.005 | 0.58 |
| **ALT** | -0.001 | 0.001 | 0.74 | 0.999 | 0.996-1.002 | 0.39 |
| **Albumin** | -0.09 | 0.03 | 12.28 | 0.91 | 0.87-0.96 | <0.001* |
| **Serum sodium** | -0.07 | 0.04 | 3.08 | 0.93 | 0.866-1.008 | 0.08 |
| **PLR** | 0.004 | 0.001 | 9.25 | 1.004 | 1.001-1.006 | 0.002* |

**Note:** *p < 0.05.

**Abbreviations:** ALT, alanine transaminase; CRP, C-reactive proteins; KD, Kawasaki disease; PLR, Platelet-lymphocyte ratio.

**Table 6** Area under the curve of variables in Model 1.

| Variables | AUC | 95%CI | p value |
| --- | --- | --- | --- |
| **Hemoglobin** | 0.669 | 0.603-0.736 | <0.001 |
| **Albumin** | 0.698 | 0.629-0.767 | <0.001 |
| **Serum sodium** | 0.641 | 0.579-0.702 | <0.001 |
| **SII** | 0.626 | 0.553-0.698 | <0.001 |

**Abbreviations:** AUC, area under curve; CI, confidence interval; SII, systemic immune inflammation index.

**Table 7** Area under the curve of variables in Model 2.

| Variables | AUC | 95%CI | p value |
| --- | --- | --- | --- |
| **Hemoglobin** | 0.669 | 0.603-0.736 | <0.001 |
| **Albumin** | 0.698 | 0.629-0.767 | <0.001 |
| **Serum sodium** | 0.641 | 0.579-0.702 | <0.001 |
| **SIRI** | 0.626 | 0.553-0.698 | 0.032 |

**Abbreviations:** AUC, area under curve; CI, confidence interval; SIRI, systemic inflammation response index.

**Table 8** Area under the curve of variables in Model 3.

| Variables | AUC | 95%CI | p value |
| --- | --- | --- | --- |
| **Hemoglobin** | 0.669 | 0.603-0.736 | <0.001 |
| **Albumin** | 0.698 | 0.629-0.767 | <0.001 |
| **Serum sodium** | 0.641 | 0.579-0.702 | <0.001 |
| **Days of IVIG at initiation** | 0.575 | 0.510-0.641 | 0.023 |
| **PIV** | 0.568 | 0.495-0.641 | 0.040 |

**Abbreviations:** AUC, area under curve; CI, confidence interval; PIV, pan-immune inflammation value.

**Table 9** Area under the curve of variables in Model 4.

| Variables | AUC | 95%CI | p value |
| --- | --- | --- | --- |
| **Hemoglobin** | 0.669 | 0.603-0.736 | <0.001 |
| **Albumin** | 0.698 | 0.629-0.767 | <0.001 |
| **NLR** | 0.626 | 0.557-0.696 | <0.001 |

**Abbreviations:** AUC, area under curve; CI, confidence interval; NLR, Neutrophil-lymphocyte ratio.

**Table 10** Area under the curve of variables in Model 5.

| Variables | AUC | 95%CI | p value |
| --- | --- | --- | --- |
| **Hemoglobin** | 0.669 | 0.603-0.736 | <0.001 |
| **Albumin** | 0.698 | 0.629-0.767 | <0.001 |
| **Days of IVIG at initiation** | 0.575 | 0.510-0.641 | 0.023 |
| **PLR** | 0.568 | 0.495-0.641 | 0.040 |

**Abbreviations:** AUC, area under curve; CI, confidence interval; PLR, Platelet-lymphocyte ratio.
